# Non-invasive neuromodulation for alleviating dyspnoea: protocol for a feasibility sham-controlled randomised trial

**DOI:** 10.1136/bmjopen-2025-103891

**Published:** 2025-07-22

**Authors:** Joël St-Pierre, Samuel Mailhot-Larouche, Geneviève Garand, Félix-Antoine Vézina, Guillaume Léonard, Christian Iorio-Morin, Simon Couillard

**Affiliations:** 1 Faculté de Médecine et des Sciences de la Santé, Université de Sherbrooke, Sherbrooke, Quebec, Canada; 2 Centre de Recherche du CHUS, Sherbrooke, Quebec, Canada

**Keywords:** Exercise, Quality of Life, Pulmonary Disease, Chronic Obstructive, Rehabilitation medicine, Adult palliative care

## Abstract

**Introduction:**

Dyspnoea affects 10% of the general population, and 12% of hospitalised patients report experiencing dyspnoea at rest. It is a common and distressing symptom experienced by people living with chronic obstructive pulmonary disease (COPD). Neuromodulation, which uses electrical stimulation to modulate neural pathways, is a validated clinical procedure offering a potential therapeutic approach. We speculate that non-invasive transcutaneous vagus nerve stimulation (tVNS) and trigeminal transcutaneous electric nerve stimulation (TENS) could improve dyspnoea management by targeting relevant neural circuits.

**Methods and analysis:**

We will conduct a feasibility cross-over trial in people with severe COPD and significant exertional dyspnoea referred for pulmonary rehabilitation. Patients will be recruited following the prerehabilitation assessment visit comprising a clinical evaluation and a maximal cardiopulmonary exercise testing on ergocycle. Subsequently, two study visits will be conducted within 2 weeks apart from each other. Eight participants will perform a submaximal constant work rate at 80% workload of the VO_2_ max, either with cervical tVNS (n=4) or trigeminal TENS (n=4). In a cross-over design, both patient groups will undergo sham and active treatment of the neuromodulation technique in a randomly assigned order. The main outcome will be feasibility, assessed by the percentage of patients who attend all visits and complete all tests. Secondary outcomes include other feasibility endpoints, the acceptability and suitability of the interventions (including an evaluation of sham as an exploratory outcome), and the incidence of adverse or undesirable events related to the procedures. Exploratory outcomes include changes in dyspnoea symptoms, measured using standardised questionnaires, such as Borg scale and the Visual Analogue Scale.

**Ethics and dissemination:**

The protocol is approved by the institutional research ethics committee of the *Centre intégré universitaire de santé et de services sociaux (CIUSSS) de l’Estrie*—CHUS, Sherbrooke, Quebec, Canada (#2025-5604) and follows *2013 Standard Protocol Items: Recommendations for Interventional Trials (SPIRIT)* guidelines. Results will be communicated in international meetings and submitted to peer-reviewed journals with respect to the *2010 CONsolidated Standards Of Reporting Trials (CONSORT)* statement for feasibility studies.

**Trial registration number:**

NCT06985628.

**STRENGTHS AND LIMITATIONS OF THIS STUDY**

- This is the first study to investigate neuromodulation for alleviating dyspnoea perception in patients with chronic respiratory disease.
- The small sample size and cross-over design are appropriate for a feasibility trial but introduce potential learning bias and limit the interpretation of the efficacy of the interventions.
- Dyspnoea perception is assessed only in severe chronic obstructive pulmonary disease patients, limiting generalisability and scales used, such as Borg and Visual Analogue Scale, may not fully reflect its complex neurological dimensions.
- The proposed sham procedure and sham-related questionnaire are not validated but were adapted from pain studies to assess the feasibility and blinding quality in this novel context.
- Although the non-invasive nature of the interventions limits the specificity of neural targeting, it offers a safe and practical foundation for testing this novel approach in standardised exercise conditions.

## Introduction

 Dyspnoea is typically described as the feeling of being unable to get enough air, defined as ‘shortness of breath’, and as the distressing experience of ‘air hunger’.^1^,^3^ This symptom is highly prevalent, affecting approximately 10% of the general population, and 12% of hospitalised patients report experiencing dyspnoea at rest.^4^,^6^ Dyspnoea is associated with various serious conditions, including pulmonary fibrosis, chronic obstructive pulmonary disease (COPD), anaemia and heart failure, where it is often the major source of patient suffering.^7^,^13^ The multifaceted nature of dyspnoea makes treating its underlying cause challenging, and current therapies, such as opioids, ventilatory support and pulmonary rehabilitation, often provide insufficient relief.^14 15^ Severe dyspnoea affects 90% of individuals with COPD in their final year of life, and nearly half do not find relief with current treatments.^16 17^ This leads to persistent, debilitating breathlessness and reduced quality of life. In Quebec, 7% of medical aid in dying requests are due to lung disease.^18^ It highlights the unmet need for innovative alternatives to better address this concerning symptom.^17^

Dyspnoea, in a complex and still not entirely well-understood manner, arises from the integration of biochemical, mechanical and neurological signals, creating a mismatch between afferent inputs and efferent pulmonary responses.^17^ This highlights that, as a sensation, dyspnoea originates from neurological pathways, presenting a potential avenue for neuromodulation.^8 9 17^ The field of neuromodulation applies precisely targeted electrical inputs to interact with the nervous system, aiming to modulate pathological signal transmission, perception and integration. Neuromodulation has gained increasing interest, given its applications in various disorders, including epilepsy (via vagus nerve stimulation), pain or angina (via spinal cord stimulation) and movement disorders (via deep brain stimulation).^17^ The similarities between dyspnoea and other sensations treated with neuromodulation, such as pain, make several neuromodulation approaches compelling options to explore dyspnoea relief.^17^ Since dyspnoea serves as an alarm signal of mismatched ventilatory demands, the goal is not to suppress this signal entirely, but rather to alleviate its excessive perception, particularly in severe cases where symptom management becomes palliative.^17^ However, understanding its feasibility and relevance requires further investigation and comprehension.

Invasive vagus nerve stimulation (VNS) is approved for treating epilepsy and depression.^19^ While transcutaneous VNS (tVNS) offers a more convenient non-invasive alternative, with growing interest for its role in respiratory pathways, it remains experimental for some uses.^20^,^24^ In theory, VNS could interfere with pulmonary sensory afferents related to dyspnoea and modulate parasympathetic signals, whose hyperactivation may worsen pulmonary symptoms.^25^,^27^

Transcutaneous electric nerve stimulation (TENS) is used in physiotherapy to treat various types of pain, such as trigeminal neuralgia, among its other uses.^28^,^31^ These treatments might be linked to the gate control theory studied in chronic pain, explaining that stimulation of particular neurologic structures may inhibit the intensity of other unpleasant afferences.^17 32 33^ Stimulation of the trigeminal nerve is supported by clinical practices such as the use of fans blowing cool air on patients’ faces, hypothesising that it activates nasal cold receptors in the V2 and V3 branches of the trigeminal nerve (cranial nerve V).^1733^,^35^ This suggests it could also be applied for other purposes, such as neuromodulation of dyspnoea.^36^

There is a recognised lack of alternative treatments for severe dyspnoea, and while opioids and other dyspnoea-directed therapies are often insufficient for alleviating this sensation, as they can be for pain management, neuromodulation may present a promising alternative.^37 38^ We hypothesise that neuromodulation techniques for relieving dyspnoea will be practical and well received by participants, and we aim to detect promising hints indicating that these methods can effectively reduce the sensation of dyspnoea. The main objective of this sham-controlled randomised trial is to evaluate the feasibility of this protocol, setting the stage for larger-scale research on the effectiveness of neuromodulation techniques for dyspnoea. The comparator in this randomised trial is a sham intervention using TENS and tVNS devices set to ineffective parameters, keeping participants blinded for a plausible study design.^39^

## Methods and analysis

The project is currently recruiting and collecting data, with expected recruitment ending by 1 December 2025, data collection by 1 February 2026 and analysis by 1 May 2026.

### Trial design and study setting

**Figure 1:**
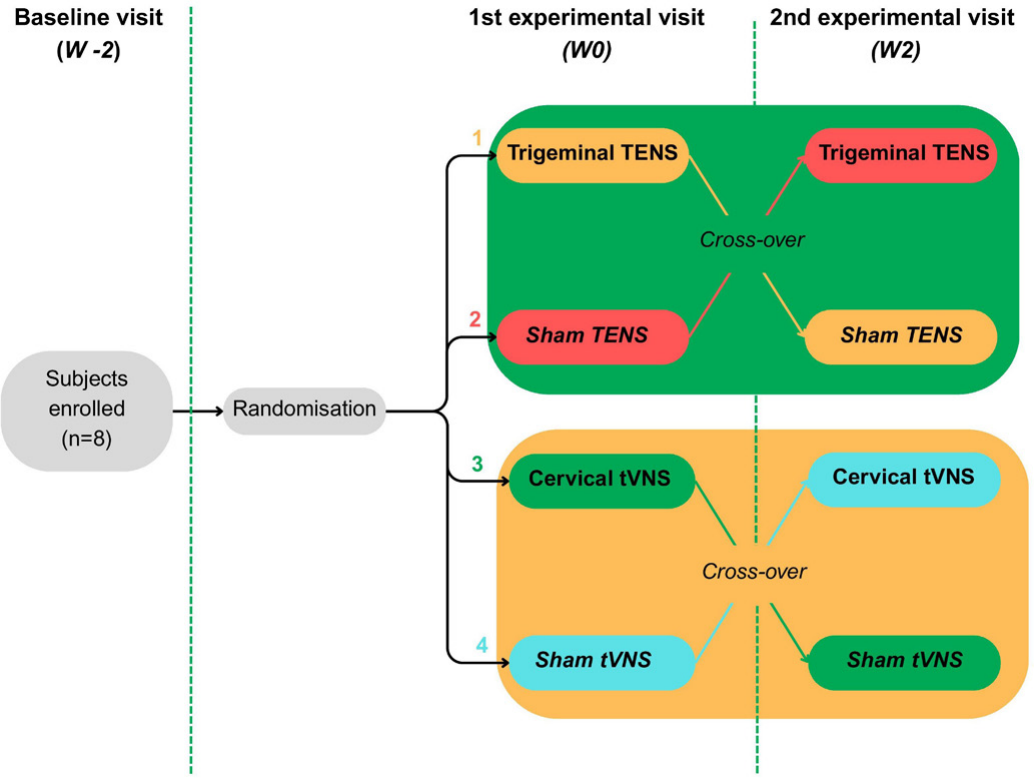
Study design of the cross-over sham-controlled feasibility trial. Description of intervention arms: (1) TENSac (visit 2 active → visit 3 sham); (2) TENSsa (visit 2 sham → visit 3 active). (3) VNSac (visit 2 active → visit 3 sham). (4) VNSsa (visit 2 sham → visit 3 active). W-2, W0 and W2 are presented as an example of ‘plausible scheduling’. An interval of 1–2 weeks will be planned between visit 2 and visit 3, whereas no specific interval (*at least 1 week*) is required between visit 1 and visit 2. TENS, transcutaneous electrical nerve stimulation; tVNS, transcutaneous vagus nerve stimulation.

We propose an observational, prospective, feasibility, cross-over trial to explore the potential of two non-invasive neuromodulation methods, tVNS and trigeminal TENS, to alleviate dyspnoea (figure 1*).* The study protocol follows the *2013 Standard Protocol Items: Recommendations for Interventional Trials (SPIRIT)* guidelines.^40^ This study employs a single-blinded format, where participants are unaware if they are receiving the sham or the real interventions, but the researchers in charge of data collection or of the analysis are not blinded. The study will be conducted at the *Centre Hospitalier Universitaire de Sherbrooke* (CHUS)’s research centre, and participants will be selected from the COPD pulmonary rehabilitation clinic at CHUS, in Quebec, Canada.

### Participants

The study includes eight adult men or women between 40 and 85 years old diagnosed with severe COPD (defined as forced expiratory volume in one second (FEV1)<50% of the predicted value (without including FEV1<20%), FEV1/forced vital capacity (FVC)<0.7 and smoking history of ≥10 pack-years, 3rd–4th grade dyspnoea (modified Medical Research Council (mMRC))^41^ for ≥6 months, COPD Assessment Test (CAT) score≥10),^42^ referred for pulmonary rehabilitation. Participants must exhibit the physical, psychological and mental capabilities to undergo submaximal constant work rate (CWR) exercise testing and answer the appropriate questionnaires. They must possess the ability to comprehend the implications of the procedures and follow-up visits and provide free informed consent.

Exclusion criteria encompass the presence of concurrent pulmonary restrictive features and neurological disorders susceptible to be influenced by neurostimulation procedures, including epilepsy, Parkinson’s disease, trigeminal neuralgia, dysautonomia and vagal disorders. Additionally, individuals with unstable arrhythmias, other cardiac instabilities or those with cardiac pacemakers, defibrillators or other metal implants, as well as those who have undergone pulmonary resection resulting in the absence of pulmonary lobe(s) or complete lung, are excluded. Furthermore, patients with COPD and other serious dyspnoeic conditions, such as lung cancer, anaemia, heart failure with reduced ejection fraction or mitochondrial dysfunction, will not be recruited for the present study. Pregnancy, active acute infections and known reactions or intolerance to tVNS or trigeminal TENS at the time of recruitment and visits also disqualify potential participants. Patients must have no moderate-to-severe exacerbations (defined as an increase in symptoms for more than 3 days requiring additional treatment (corticosteroids or antibiotics) compared with the usual regimen or hospitalisation)^43^ for at least 1 month before baseline visit and the first experimental visit (W0 visit). Participants who are randomised but experience an exacerbation between the first and second (W2) experimental visits will not complete the W2 visit. However, if the patient remains eligible and still consents, they may be re-randomised into the trial 1 month after the exacerbation, under the same pre-W0 visit conditions, and begin again from the W0 visit (table 1).

**Table 1:** Participant inclusion and exclusion criteria

Inclusion criteria	Exclusion criteria
40–85 years	Neurological conditions: Epilepsy Parkinson’s disease Trigeminal neuralgia Dysautonomia Vagal disorders
FEV1 between 20% and 50% of the predicted value	Other medical conditions: Restrictive pulmonary syndrome Unstable arrhythmias Anaemia Heart failure with reduced ejection fraction Mitochondrial dysfunction Lung cancer
FEV1/FVC ratio<0.7	Pacemaker, defibrillators or other metallic implants
Smoking history of ≥10 pack-years	Lung resection surgery resulting in the absence of a lobe or a complete lung
Grade 3–4 on mMRC for ≥6 months	Pregnant women
CAT≥10	Active acute infection
Referred for pulmonary rehabilitation	Past reactions or intolerances to VNS or TENS
Physically, mentally and psychologically capable of undergoing CWR and answering questionnaires	Moderate-to-severe exacerbations in the last 1 month before the study visits

CAT, COPD Assessment Test; COPD, chronic obstructive pulmonary disease; CWR, constant work rate; FEV1, forced expiratory volume in one second; FVC, forced vital capacity; mMRC, modified Medical Research Council Scale; TENS, transcutaneous electrical nerve stimulation; VNS, vagus nerve stimulation.

### Study interventions

In the first arm (TENS), participants undergo submaximal CWR with the addition of a non-invasive trigeminal TENS, administered or expertly guided by a physiotherapy expert using the methods of this device. The TENS technique uses electromagnetic waves to modulate nerve transmission transcutaneously. When electrodes are placed on the skin, approximately over the temporomandibular joint, it is well established that they can modulate trigeminal nerve activity with the appropriate settings. We propose an initial configuration with a pulse duration of 60 µs at a frequency of 100 Hz, but the parameters may be adjusted during preliminary tests of the devices and for each patient as needed. For the sham trigeminal TENS, we suggest informing patients that two different modes of stimulation will be used, one of which will feel stronger than the other. This allows us to activate the device during the intervention and adjust it to a level where the skin experiences a mild tingling sensation. This stimulation will remain below the threshold of the gate control theory, being clinically ineffective, without explicitly informing the patients of this detail, in accordance with the attested consent form.^44^,^47^

In the second arm (tVNS), participants undergo submaximal CWR with the addition of cervical tVNS (*gammaCore Sapphire D Device, electroCORE*: Food and Drug Administration cleared^48 49^ and approved by *Health Canada*^50^). This technique will be applied or expertly guided by a neurosurgeon specialised in functional neurosurgery. The cervical tVNS device is applied manually to the front of the neck, near the trachea, to modulate afferent and efferent vagal nerve activity. As the *gammaCore* device is indicated and configured for the treatment of migraines, some parameters, such as a 5000 Hz pulse at 25 Hz, are fixed, while the stimulation voltage (limited to a peak of 24 V), and current (mA) remain adjustable and variable. This low-voltage VNS may be more advantageous for respiratory modulation.^51^

The effectiveness threshold, related to pain modulation, in terms of amplitude of the electrostimulation, depends on the patient. Then, for both tVNS and trigeminal TENS, the amplitude will be adjusted to reach a threshold where the patient perceives a strong stimulation, experiencing paraesthesia on the trigeminal area, while remaining comfortable for them.^52^ As an exploratory study, the initial stimulation parameters follow standard settings used for the device in their currently indicated conditions, but frequency, mode of stimulation (eg, burst, continuous), intensity and electrode placement remain open to investigation to expand knowledge in the field of dyspnoea neuromodulation. For the sham tVNS, the device will follow a similar procedure as the sham TENS. For both the sham and intervention, light and sound confounding stimuli on the devices will be used to enhance blinding efficiency and minimise the placebo effect in our comparative analysis.^53^ For the elaboration and application of the conceptualised sham-TENS and sham-tVNS by our research team, we thoroughly follow the *Template for Intervention Description and Replication (TIDieR)-Placebo reporting guideline*.^54^

### Outcomes

#### Primary (feasibility assessment)

1. Sample study completion proportion: percent of recruited patients attending all visits and completing all tests.

#### Secondary feasibility assessment

1. Acceptability of this study procedures (CWR, TENS, tVNS, etc) assessed by Likert score at the end of the last visit.
2. Recruitment results: percent of recruited patients on the target sample size (which is 2 × n=4), and on the number of patients approached for screening.
3. Numerical and descriptive occurrence of adverse effects and undesirable events throughout the complete study period, as reported by the patients and investigated by the researchers.

#### Exploratory

1. Absolute change from baseline in maximum Borg score with a minimal clinically important difference (MCID)=1; and Borg score slope during test (measured every minute during CWR).
2. Absolute change from baseline in pedalling time at the same pace and workload.
3. Other statistical analysis of the data collected (questionnaires, test parameters, observations, etc) from participants to investigate the potential effect of neurostimulation (tVNS and TENS) on dyspnoea management.
4. Assessment, through observational notes, of the ease of use and portability of VNS and TENS devices for patients with chronic pulmonary diseases.
5. Assessment of the convenience and practicality of integrating VNS and TENS into their daily routines by patient-reported feedback (by Likert score and by free comments provided).
6. Blinding assessment of the sham will be based on expectation and efficacy questions during visits and the conceptualised sham questionnaire at the end of the last visit.

### Schedule of visits

Although this is a feasibility study focusing on recruitment, completion, adverse effects and acceptability (using a Likert questionnaire (online supplemental appendix 1), our overarching research aims to explore the impact of dyspnoea in individuals by using the submaximal CWR^55 56^ at 80% of VO_2_ max to experimentally provoke dyspnoea symptoms and evaluate the effects of our proposed relieving techniques. According to the *American Thoracic Society 2019* standards,^57^ spirometry will be employed to evaluate pulmonary function, encompassing key parameters such as FEV1, FVC and FEV1/FVC. Basic vital signs, including oxygen saturation (SpO_2_), heart rate (HR), arterial blood pressure (BP), respiratory rate (RR) and temperature (T°), will also be measured to ensure the safety of the patients and to support the study. Subjective dyspnoea assessments will be carried out using the Borg scale (0–10) (online supplemental appendix 2),^58 59^ a 100 mm Visual Analogue Scale (VAS) for dyspnoea (online supplemental appendix 3) complemented by the mMRC dyspnoea scale for individuals with COPD (online supplemental appendix 4), the CAT questionnaire (online supplemental appendix 5), *St George’s Respiratory Questionnaire (SGRQ)* (online supplemental appendix 6) and the time to return to initial Borg Score serves as a measure of recovery following the exercise test. Data on rehabilitation performance will include measurements of workload, VO_2_ max, pedalling time and breathing and cardiac reserves. To confirm (yes or no) whether the baseline visit reached maximal effort at VO_2_ max, the criteria for maximal effort from the *European Respiratory Society (ERS) statement on the standardisation of cardiopulmonary exercise testing (CPET) in chronic lung diseases* will be applied*.*^60^ The quality of blinding with the proposed sham will also be assessed (online supplemental appendix 7). Other questions will assess participants’ expectations and perceived intervention efficacy during each exercise test using a Numeric Rating Scale (NRS) (online supplemental appendix 8). A short questionnaire will evaluate the anxiety, depression and catastrophisation related to breathlessness (online supplemental appendix 9). All the data expected to be collected are detailed in online supplemental tables 1 and 2*.*

Throughout the entire duration of the study, participants’ complete medication regimen will be assessed, and their medication must remain stable during the trial. As part of the pulmonary rehabilitation programme, the eight participants will undergo a medically advised first CPET at VO_2_ max following the data measurement procedures of the study. The Borg scale will be evaluated before the test, every 3 min during the test and at the end of the test. For the baseline visit, only SpO_2_ will be measured before and after CPET. Additionally, spirometry parameters, including FEV1, FVC and FEV1/FVC, will be measured before the test. Data on rehabilitation performance, such as VO_2_ max, breathing and cardiac reserve and the assessment of maximal effort testing, will be useful for the setting of the next two visits. This baseline CWR is crucial as it sets the parameters for the next two experimental visits and as it accounts for the effects of adaptation to the physical test (learning effect), ensuring that any observed changes in subsequent tests are not merely due to physiological adaptations or normal variations that occur in every test conducted under this protocol.

At least 1 week later, at the beginning of this first experimental visit, demographic data and medical records (confirmed via data entry form), the SGRQ and the Psychological Components Questionnaire will be administered. At this point, participants will receive either a cervical or facial neurostimulator, delivering cervical tVNS or trigeminal TENS. Then, they will undergo a submaximal CWR at 80% of VO_2_ max, with the intervention or the sham, and with the same data measurement of the baseline CWR with the addition of more dyspnoea evaluation questionnaires. These include the Borg scale assessed every minute during the test, the mMRC and CAT questionnaires administered before the test, and the VAS recorded before and after CWR. Participants’ expectations regarding the efficacy of the intervention will be assessed at rest, both with and without stimulation. At the end of the protocol, stimulation and perceived efficacy will be evaluated to gather additional subjective data on relief perception and the quality of the sham. Additionally, the time taken to return to the initial Borg score at rest following CWR will be measured. Vital signs, including SpO_2_, HR, BP, RR and T° will also be monitored throughout the entire duration of the test (except BP and T° that are only assessed before CWR).

Within 2 weeks after the first experimental visit, those who received an intervention (tVNS or trigeminal TENS) will receive the sham, and those who received the sham will receive an intervention. This switch occurs according to the randomisation process of the cross-over group assignment (figure 1). They all undergo a final submaximal CWR, with data collected in the same sequence during the first experimental visit.

In case of technical issues or other concerns affecting the validity of the test or the data collected during one of the visits, an additional visit (W4) could be added after the last experimental visit. If this visit takes place, it will follow the same exercise testing and intervention protocols as the two preceding experimental visits.

If patients experience significant discomfort or adverse effects during the test or interventions, they should immediately inform the healthcare provider so that appropriate measures can be taken to reassure and manage the patient effectively, including possibly stopping the procedure if necessary. The exercise test termination criteria, established by the *American Thoracic Society*, will be followed to ensure participant safety.^56^ Otherwise, common short-term side effects will be assessed frequently, with a minimum of one evaluation at the end of each experimental visit using a standardised questionnaire designed for the interventions of the study. This approach aims to promptly identify and screen for any undesirable effects experienced by participants (online supplemental appendix 10).

Study visits will be completed before starting or before completing the 12-week rehabilitation programme, with a minimal interval to allow recovery after exercise testing while limiting external influences on exercise tolerance assessments. The schedule for data collection of each variable is detailed in online supplemental table 3*.*

### Sample size and feasibility

Eight patients (n=8) from the pulmonary rehabilitation programme of the *Centre intégré universitaire de santé et de services sociaux (CIUSSS) de l’Estrie*—CHUS will be recruited for a feasibility study to evaluate differences between a sham group and an intervention group. The primary aim is to assess the viability of the protocol and the feasibility of completing outcome measurements. This study is not designed to determine the efficacy of the intervention, nor to tease out responder groups or sex differences; rather, it aims to inform future research in the field of neuromodulation.

The operational feasibility of using non-invasive neuromodulation techniques is supported by Health Canada’s approval of tVNS *gammaCore* devices and TENS, underscoring the potential for these therapies to be adapted for clinical uses. The tVNS device is a technique mastered by CI-M, and the TENS is mastered by GL’s team.

### Recruitment strategy

Potential participants meeting the inclusion and exclusion criteria will be recruited directly from the pulmonary rehabilitation programme of Estrie, Quebec, Canada. Using prescreening of the programme’s clinical schedule by authors JS-P and SM-L or respiratory therapist GG, the clinical team will be alerted to potential candidates and approach these independently of the research team. Interested participants will be scheduled for the first research visit before completing the rehabilitation programme, where a free and informed written consent will be obtained.

### Assignment of interventions

At visit 1, participants will be randomised, using randomisation function of REDCap, to one of four groups for subsequent study procedures: TENSac (visit 2 active → visit 3 sham) or TENSsa (visit 2 sham → visit 3 active), VNSac (visit 2 active → visit 3 sham) or VNSsa (visit 2 sham → visit 3 active), in a 1:1:1:1 ratio through the randomisation function of REDCap. A non-blinded investigator will run the randomisation and allocation process. Given our small sample size (n=8), no stratification will be applied for randomisation.

However, in a future randomised controlled trial, the randomisation should be minimised for FEV_1_ (<35% or ≥35% predicted), sex (one female participant (n=1) and one male participant (n=1), for a total of two participants per group arm (n=2) of the randomisation) and the number of severe exacerbations in the preceding 12 months (0 or ≥1 events).

### Statistical analyses

Descriptive analysis will be conducted for the primary objectives to evaluate the feasibility parameters. For the exploratory objective concerning the alteration in dyspnoea perception and pulmonary parameters, several statistical methods will be employed. The Borg slope will be analysed using a linear mixed model, which accounts for both fixed and random effects, allowing for the evaluation of changes over time within individuals. Paired sample t-tests will compare parameters with and without the interventions, determining if there is a significant difference. The χ^2^ test will assess the association between categorical variables.

### Ethics and dissemination

The protocol is approved by the research ethics committee of the CIUSSS de l’Estrie—CHUS, Sherbrooke, Quebec, Canada (#2025-5604). Informed consent or assent will be explained and obtained by JS-P, F-AV, GG or SM-L, from patients who meet the inclusion and exclusion criteria at the baseline visit.

It follows the *2013 SPIRIT* guidelines for a clinical trial protocol.^40^ Results will be communicated in international meetings and submitted to peer-reviewed journals in a format respecting the *CONsolidated Standards Of Reporting Trials (CONSORT) 2010* statement.^61^

#### Data and sample management

Demographic and medical-record data regarding patients will be collected for patient enrolment based on inclusion and exclusion criteria and to analyse correlations between feasibility outcomes and patient characteristics. This data will only be accessible through specific computers with authorised permission to access the *Ariane* system at CHUS. Throughout each data collection session for the study’s tests, comprehensive notes will be written manually on paper clinical notes, and standardised scales and clinical research forms will be completed by hand. The data will then be transcribed into a *REDCap* project for optimised data management.^62 63^ No data monitoring committee was deemed necessary, as the risks associated with the study’s non-invasive procedures are considered minimal. The interventions, tVNS and TENS, involve clinically approved devices with a well-established safety profile and no significant risk.

## Discussion

This study proposes a sham-controlled randomised cross-over trial to assess the feasibility of using tVNS and trigeminal TENS to alleviate dyspnoea in patients with severe COPD, in which they will undergo submaximal CWR exercise test at 80% of VO_2_ max receiving either the active intervention or a sham treatment, based on cross-over randomisation. The main goal of the study is to assess the feasibility outcomes, primarily the proportion of completion, along with the acceptability of procedures, recruitment success and occurrence of adverse events, in order to guide the design of future large-scale studies evaluating the potential of neurostimulation to manage dyspnoea.

There are limitations to the study proposal. First, the small sample size implies that we will likely be unable to conclude on any therapeutic efficacy of the interventions. In relation to the study design, the cross-over design, where participants undergo both the intervention and the sham procedure, could also present a limitation. This approach carries the risk of unblinding the trial, which could lead to observed effects being heavily influenced by the placebo effect. However, this is appropriate for a feasibility trial. Second, the proposed sham procedure and sham-related questionnaires have not been validated, further reinforcing the need for a first feasibility study prior to full clinical trial. Third, dyspnoea will be monitored using the Borg scale and VAS during a submaximal CWR test at 80% of VO_2_ max, complemented by additional questionnaires to assess pulmonary perceptions over time, including at rest. We acknowledge that the perception of dyspnoea is difficult to quantify, as we have previously shown that the Borg scale poorly correlates with objective metrics such as FEV_1_-quantified bronchoconstriction.^64^ Finally, we have limited the study population to COPD, whereas neuromodulation may potentially be useful for dyspnoea related to a variety of conditions. In summary, this first feasibility trial will allow us to strengthen our methods for future clinical trials.

Neuromodulation has expanded significantly in recent decades, with numerous approaches being explored to modulate symptoms of various burdensome diseases, and not always purely of neurological nature. This study aligns with the growing interest in symptom modulation, but it takes a less-explored avenue.^38^ To our knowledge, a clinical approach to evaluate the effects of neurostimulation on the perception of dyspnoea has not been previously explored in the scientific literature.

In conclusion, our non-invasive neuromodulation approach with tVNS and trigeminal TENS aims to explore the roles of these nerves and demonstrate that it is reasonable to study the impact of neuromodulation on dyspnoea. Developing the field of neuromodulation for novel applications in dyspnoea has the potential to lead to a wider research programme, which could significantly advance dyspnoea management and ultimately improve patients’ quality of life and functional capacity regarding a devastating symptom experienced across multiple diseases.

## Supplementary material

10.1136/bmjopen-2025-103891 online supplemental file 1
