# Supplementary material for: Non-invasive neuromodulation for alleviating dyspnoea: protocol for a feasibility sham-controlled randomised trial
Source: BMJ Open. 2025 Jul 22;15(7):e103891. doi: 10.1136/bmjopen-2025-103891 (PMC12306373; doi:10.1136/bmjopen-2025-103891)
Supplement: online supplemental file 1 [file bmjopen-15-7-s001.docx]

**ALLEVATING DYSPNEA WITH NON-INVASIVE NEUROMODULATION: A FEASIBILITY SHAM-CONTROLLED RANDOMIZED TRIAL**

**SUPPLEMENTARY MATERIAL**

Table of Contents

***Supplementary Tables***

[TABLE 1. Demographic data and medical records 2](#_Toc190267124)

[TABLE 2. Experimental data 3](#_Toc190267126)

[TABLE 3. Schedule of activities 5](#_Toc190267127)

[APPENDIX 1. Likert score for acceptability of the study procedures 6](#_Toc190267128)

[APPENDIX 2. BORG questionnaire 9](#_Toc190267130)

[APPENDIX 3. Visual analogue scale of dyspnea symptoms 10](#_Toc190267131)

[APPENDIX 4: Modified Medical Research council dyspnea scale (mMRC) 11](#_Toc190267132)

[APPENDIX 5: COPD Assessment test (CAT) 12](#_Toc190267133)

[APPENDIX 6: St George's Respiratory Questionnaire (SGRQ) 13](#_Toc190267134)

[APPENDIX 7. Sham evaluation questionnaire 13](#_Toc190267136)

[APPENDIX 8. Numeric Analogue Scales for Intervention Perception 14](#_Toc190267137)

[APPENDIX 9. Psychological Components Questionnaire 15](#_Toc190267138)

[APPENDIX 10. Clinical Undesirable Effects Screening Questionnaire 16](#_Toc190267139)

###

### TABLE 1. Demographic data and medical records

| Variable | Description | Source | Scale/unit | Analysis method |
| --- | --- | --- | --- | --- |
| Age |  | Initially assess by W0 visit for baseline submaximal CWR | Year | Descriptive:  Mean and standard deviation |
| Sex at birth | Male or female |  |  |  |
| Gender | Male, female, transgeder, two-spirit, other |  |  |  |
| Ethnic group | White, south-Asian, Chinese, Black, Philippino, Arabic, latin-american, south-eastern Asian, Korean, Japanese, other |  |  |  |
| Height |  |  | cm |  |
| Weight |  |  | kg |  |
| BMI |  |  | Kg/m2 |  |
| Work/occupation |  |  |  |  |
| Relevant medical records | Comorbidities (Pulmonary, cardiovascular, renal, neurologic disease), past interventions, hospitalisation, allergies | Access to ARIANE medical records of the patients and assess by questioning the participant before W0 | Listed |  |
| Charlson index | Prediction of 10-year survival. |  | % |  |
| Medication | Past and actual (bronchodilator, corticosteroid, antibiotics, etc.) |  | Listed |  |
| COPD history | Evolution, year of the diagnosis, number of exacerbations and medical visits for COPD, consideration of medical assistance in dying |  |  |  |
| Consumption | Tobacco, alcohol, drug |  |  |  |

### ** Demographic data will be collected for statistical analysis to assess tendencies related to the patients recruited and those who have completed every test.*

### TABLE 2. Experimental data

| Variable | Description | Source | Scale/unit | Analysis method |
| --- | --- | --- | --- | --- |
| Acceptability of study procedures | Likert score ([Appendix 1](#_APPENDIX_1._Likert)) | Assess at the end of W2 visit | Score out of 45 pts for the analysis of protocol acceptability according to the participants | Descriptive |
|  | Adverse effects and undesirable events  ([Appendix 7](#APPENDIX7_Clinical)) | Assess during every visit (W-2, W0 and W2) | Observational notes | Descriptive |
|  | Participants’ perception (Pain and other symptoms and sensations) | Assess during every visit (W-2, W0 and W2) | Observational notes and grade scale of pain intensity (or other discomfort) from 0-10 | Descriptive |
| Recruitment results | % of patients recruited according to the target | Assess before W0 |  | Number of patients recruited/2*(n=4) |
|  | % of patients recruited according to the total approached | Assess before W0 |  | Number of patients recruited/Total of patients approached |
| Sample study completion proportion | % of patients attending all visits and completing all tests | Assess after W2 |  | Number of patients attending all visits and completing all tests/Number of patients recruited |
| Adverse effects and undesirable events | Effects directly regarding the interventions and sham | Assess during every visit (W-2, W0 and W2) | Observational notes | Descriptive |
| Spirometry parameters | FEV1, FVC, FEV1/FVC | Assess before submaximal CWR | L, L/s, LPM, and/or % predicted | Predicted values as per Race-neutral Global Lung Initiative |
| Vital signs | SpO_2_, HR, BP, RR, T˚ | Assess during the full period of submaximal CWR (SpO_2_ and T˚ is assessed only at rest before CWR) | %, bpm, mmHg, unit, ˚C |  |
| Workload |  | Assess during every CWR | W and % of VO2max workload | Pedaling time |
| VO2 max |  | Assess during every CWR | L/min , mL/kg/min, % predicted, % maximum | Mean and peak values during submaximal endurance CWR |
| Pedaling time |  | Assess during every CWR (W0, W2) | Time (s) | MCID : 45-106 s |
| Breathing and cardiac reserves |  | Assess during every CWR | % | Descriptive |
| Assessment of maximal effort testing | Based on ERS statement | Assess at baseline visit (W-2) | Binary qualitative (Yes, No) | Descriptive |
| Reason for test termination | Legs, dyspnea, other | Assess at the end of CWR | Observational | Qualitative |
| Dyspnea perception |  | | Semi-quantitative | MCID |
|  | Borg scale | Assess before, every minute during submaximal CWR until resolution of Borg score (maximum 10 minutes after). | 0-10 points | 1 point |
|  | VAS | Assess at rest before and after the CWR (W0 and W2) | 0-100 mm | 17 mm |
|  | mMRC dyspnea scale | Assess at the beginning of each visit | 0-4 grades | 1 grade |
|  | CAT |  | 0-40 points | 2 points |
|  | St George's Respiratory Questionnaire (SGRQ) | Assess once at the beginning of W0 visit. | % (0,00-100)  Total and subtotal (symptoms, activities, impacts) | St George's Respiratory Questionnaire (SGRQ) |
|  | Time to return to initial Borg Score after CWR | Assess after CWR (W0 and W2 | Time (s) | Descriptive |
|  | NRS for the expectations, stimulation and efficacy  ([Appendix 8](#_APPENDIX_8._Numeric)) | Assess at the beginning, at rest with the stimulation, and at the end of the visit. (W0 and W2) | NRS 0 to 10 | Descriptive |
|  | Psychological Components Questionnaire (PCQ) : anxiety, depression, catastrophization  ([Appendix 9](#_APPENDIX_9._Psychological)) | Assess once at the beginning of W0 visit. | 0 to 10 (intensity)  0 to 4 (frequency) | Descriptive |
| Sham evaluation | Questionnaire to participants to scope their perception of whether they received the real or sham intervention.  [Appendix 2](#_APPENDIX_2._Sham) | Assess at the end of W2 visit |  | Descriptive |

### TABLE 3. Schedule of activities

| **Procedure** | **Clinical visit for pulmonary rehabilitation**  **(W-2)*** | **Intervention period** | | **Notes** |
| --- | --- | --- | --- | --- |
|  |  | W0 | W2 |  |
| Informed consent | X |  |  |  |
| Inclusion/exclusion criteria | X | X | X |  |
| Demographic data | X* | X |  |  |
| Relevant medical records | X* | X |  |  |
| Medication review | X* | X | X |  |
| Feasibility data (acceptability (Likert score), %recruitment, %completion, adverse and undesirable events) |  | X | X | Likert score and %recruitment will only be assessable after W2 |
| Spirometry measurements, vital signs, pedaling time, exercise test, rehabilitation performance data | X  (VO2Max) | X  (submax CWR) | X  (submax CWR) |  |
| Dyspnea perception scale (Borg scale, mMRC dyspnea scale, VAS, CAT, Time to return to initial Borg score, NRS, PCQ ) | X  (Borg scale, CAT and mMRC) | X  (SGRQ, PCQ assessed once at this visit) | X | -Borg scale: before, q 1 min during CWR until 10 min after  -VAS : before and after CWR  -mMRC and CAT: Only assess once before CWR  - NRS : At rest, with and without stimulation, and at the end. |
| Sham evaluation |  |  | X |  |

* The clinical visit data will be extracted after informed consent has been obtained.

## APPENDIX 1. Likert score for acceptability of the study procedures

## (Score on 45 pts)

1. **Effectiveness: How effective do you believe these interventions are on dyspnea?**

1: Not effective at all

2: Slightly effective

3: Moderately effective

4: Quite effective

5: Very effective

1. **Impact on Quality of Life: How much do you think these interventions improve the quality of life for you and generally for severely dyspneic patients?**

1: No improvement at all

2: Slight improvement

3: Moderate improvement

4: Significant improvement

5: Very serious impact

1. **Safety: How concerned are you about the safety of these interventions?**

1: Not concerned at all

2: Slightly concerned

3: Moderately concerned

4: Very concerned

5: Completely concerned

1. **Side Effects: How concerned are you about the potential side effects of these interventions?**

1: Not concerned at all

2: Slightly concerned

3: Moderately concerned

4: Very concerned

5: Completely concerned

1. **Acceptability: How acceptable do you use these interventions to treat severely dyspneic patients like you?**

1: Not acceptable at all

2: Slightly Acceptable

3: Moderately acceptable

4: Very acceptable

5: Completely acceptable

1. **Patient Compliance: How confident are you that patients will comply with theses interventions protocols?**

1: Not confident at all

2: Slightly confident

3: Moderately confident

4: Very confident

5: Completely confident

1. **Accessibility: How accessible do you find these interventions are for the general population (regarding the availability, the cost, the professional involved, etc.)?**

1: Not accessible at all

2: Slightly accessible

3: Relatively accessible

4: Well accessible

5: Very accessible

1. **Awareness: How aware are you of the latest advancements in neuromodulation technology related to your comprehension in management dyspnea**?

1: Not aware at all

2: Slightly aware

3: Moderately aware

4: Well-aware

5: Very aware

1. **Importance: How important do you believe these interventions are in the future of dyspnea management?**

1: Not important at all

2: Slightly important

3: Moderately important

4: Important

5: Very important

## APPENDIX 2. BORG questionnaire

## APPENDIX 3. Visual analogue scale of dyspnea symptoms

## APPENDIX 4: Modified Medical Research council dyspnea scale (mMRC)

## APPENDIX 5: COPD Assessment test (CAT)

##

## APPENDIX 6: St George's Respiratory Questionnaire (SGRQ)

**PART 1**

**Questions about how much chest problem you have had over the past 3 months.**

*Please checkmark (X) one box for each question:*

|  | Most days of the week | Several days a week | A few days a month | Only chest infections | Not at all |
| --- | --- | --- | --- | --- | --- |
| 1. Over the past 3 months, I have coughed: |  |  |  |  |  |
| 1. Over the past 3 months, I have brought up phlegm (sputum): |  |  |  |  |  |
| 1. Over the past 3 months, I have had shortness of breath: |  |  |  |  |  |
| 1. Over the past 3 months, I have had attacks of wheezing: |  |  |  |  |  |
| 1. During the past 3 months, how many severe or very unpleasant attacks of chest problem have you had? | more than 3 attacks  3 attacks  2 attacks  1 attack  no attacks  | | | | |
| *(Go to question 7 if you had no severe attacks)*   1. How long did the worst attack of chest problem last: | a week or more  3 days or more  1 or 2 days  Less than a day  | | | | |
| 1. Over the past 3 months, in an average week, how many good days(with little chest problem) have you had: | No good days  1 or 2 good days  3 or 4 good days  Nearly every day was good  Every day was good  | | | | |
| 1. If you have a wheeze, is it worse in the morning: | No  Yes  | | | | |

**PART 2**

*Please checkmark (X) one box for each question:*

SECTION 1

How would you describe your chest condition?

The most important problem I have

Causes me quite a lot of problems

Causes me a few problems

Cause me no problem

If you have ever had paid employment.

My chest problem made me stop work altogether

My chest problem interferes with my work or made me change my work

My chest problem does not affect my work

SECTION 2

**Questions about what activities usually make you feel breathless these days.**

| *For each item, please checkmark (X) the box as it applies to you these days:* | **TRUE** | **FALSE** |
| --- | --- | --- |
| Sitting or lying still |  |  |
| Getting washed or dressed |  |  |
| Walking around at home |  |  |
| Walking outside on the level |  |  |
| Climbing up a flight of stairs |  |  |
| Climbing hills |  |  |
| Playing sports or games |  |  |

SECTION 3

**Some more questions about your cough and breathlessness these days.**

| *For each item, please checkmark (X) the box as it applies to you these days:* | **TRUE** | **FALSE** |
| --- | --- | --- |
| My cough hurts |  |  |
| My cough makes me tired |  |  |
| I am breathless when I talk |  |  |
| I am breathless when I bend over |  |  |
| My cough or breathing disturbs my sleep |  |  |
| I get exhausted easily |  |  |

SECTION 4

**Questions about other effects that your chest problem may have on you these days.**

| *For each item, please checkmark (X) the box as it applies to you these days:* | **TRUE** | **FALSE** |
| --- | --- | --- |
| My cough or breathing is embarrassing in public |  |  |
| My chest problem is a nuisance to my family, friends or neighbours |  |  |
| I get afraid or panic when I cannot get my breath |  |  |
| I feel that I am not in control of my chest problem |  |  |
| I do not expect my chest to get any better |  |  |
| I have become frail or an invalid because of my chest |  |  |
| Exercise is not safe for me |  |  |
| Everything seems too much of an effort |  |  |

SECTION 5

**Questions about your medication. *If you are taking no medication go straight to Section 6.***

| *For each item, please checkmark (X) the box as it applies to you these days:* | **TRUE** | **FALSE** |
| --- | --- | --- |
| My medication does not help me very much |  |  |
| I get embarrassed using my medication in public |  |  |
| I have unpleasant side effects from my medication |  |  |
| My medication interferes with my life a lot |  |  |

SECTION 6

**These are questions about how your activities might be affected by your breathing.**

| *For each item, please checkmark (X) the box as it applies to you because of your breathing* | **TRUE** | **FALSE** |
| --- | --- | --- |
| I take a long time to get washed or dressed |  |  |
| I cannot take a bath or shower, or I take a long time |  |  |
| I walk slower than other people, or I stop for rests |  |  |
| Jobs such as housework take a long time, or I have to stop for rests |  |  |
| If I walk up one flight of stairs, I have to go slowly or stop |  |  |
| If I hurry or walk fast, I have to stop or slow down |  |  |
| My breathing makes it difficult to do things such as climbing up hills, carrying things up stairs, light gardening such as weeding, dancing, playing bowls or golf |  |  |
| My breathing makes it difficult to do things such as carrying heavy loads, digging the garden or shovelling snow, jogging or walking at 8 kilometres per hour, playing tennis or swimming |  |  |
| My breathing makes it difficult to do things such as very heavy manual work, running, cycling, swimming fast or playing competitive sports |  |  |

SECTION 7

**We would like to know how your chest problem usually affects your daily life.**

| *For each item, please checkmark (X) the box as it applies to you because of your breathing* | **TRUE** | **FALSE** |
| --- | --- | --- |
| I cannot play sports or games |  |  |
| I cannot go out for entertainment or recreation |  |  |
| I cannot go out of the house to do the groceries |  |  |
| I cannot do housework |  |  |
| I cannot move far from my bed or chair |  |  |

*Here is a list of other activities that your chest problem may prevent you doing (you do not have to checkmark these, they are just to remind you of ways in which your breathlessness may affect you):*

*Going for walks or walking the dog, doing things at home or in the garden, sexual intercourse, going out to church or place of entertainment, going out in bad weather or into smoky rooms, visiting family or friends or playing with children*

*Please write in any other important activities that your chest problem may stop you doing: ......................................................................................................................................................................................................................................................................................................................................................................................*

*...........................................................................................................................................................................................*

Now, would you checkmark the box (one only) which you think best describes how your chest affects you:

It does not stop me doing anything I would like to do

It stops me doing one or two things I would like to do

It stops me doing most of the things I would like to do

It stops me doing everything I would like to do

## APPENDIX 7. Sham evaluation questionnaire

**1. Over the two visits, you experienced two different stimulation modes from the device. If you had to choose, which intervention would provide the most relief for your breathlessness?**

1) Intervention of the 1st visit □

2) Intervention of the 2nd visit (today) □

3) Both interventions had similar effects □

**2. On a scale of 1 to 5, how confident are you in the effectiveness of the intervention you chose in question 1?**

1: Not at all confident

2: Slightly confident

3: Moderately confident

4: Very confident

5: Completely confident

**3. What reason(s) led you to choose the intervention in question 1?**

*If option 3 was selected in question 1, skip to question 7.*

**4. What aspects of the intervention procedure led you to believe that one intervention might be less effective than the other?**

Noise/sound □

Vibrations □

Sensation on the face or neck □

Visual stimuli (example : light) □

Device application (placement, techniques, etc.) □

Other : ____________________________________________________

**5. Did the investigators (research professionals) provide any hints that led you to believe one intervention might be more effective than the other?**

YES □

NO □

IF YES, could you specify :

**6. How confident are you in the effectiveness of the intervention you DID NOT choose in question 1?**

1: Not at all confident

2: Slightly confident

3: Moderately confident

4: Very confident

5: Completely confident

**7. What are the positive effects or aspects about the interventions?**

**8. What are the negative effects or limitations about the interventions?**

**9. After experiencing the types of stimulation during your two visits, what recommendations or improvements would you suggest regarding the application of our intervention?**

## APPENDIX 8. Numeric Rating Scales for Intervention Perception

For stimulation intensity: On a scale of 0 to 10, how intense was the stimulation you received (on the face or neck)?

| **0** | **1** | **2** | **3** | **4** | **5** | **6** | **7** | **8** | **9** | **10** |
| --- | --- | --- | --- | --- | --- | --- | --- | --- | --- | --- |
| No sensation |  |  |  |  |  |  |  |  |  | At the limit of painful |

For participant expectations regarding effectiveness: On a scale of 0 to 10, what are your expectations regarding the effectiveness of our intervention in relieving breathlessness?

| **0** | **1** | **2** | **3** | **4** | **5** | **6** | **7** | **8** | **9** | **10** |
| --- | --- | --- | --- | --- | --- | --- | --- | --- | --- | --- |
| The intervention will not be helpful |  |  |  |  |  |  |  |  |  | The intervention will completely relieve breathlessness |

For the perception of relief (at the end of the visit): After today's experimental protocol (under stimulation from our device), using this scale from 0 to 10, how would you rate the relief of your breathlessness from the intervention you received today?

| **0** | **1** | **2** | **3** | **4** | **5** | **6** | **7** | **8** | **9** | **10** |
| --- | --- | --- | --- | --- | --- | --- | --- | --- | --- | --- |
| No change |  |  |  |  |  |  |  |  |  | Maximum relief of breathlessness |

## APPENDIX 9. Psychological Components Questionnaire

1. To what extent do you feel anxious about the breathlessness associated with your health condition?

| **0** | **1** | **2** | **3** | **4** | **5** | **6** | **7** | **8** | **9** | **10** |
| --- | --- | --- | --- | --- | --- | --- | --- | --- | --- | --- |
| None at all |  |  |  |  |  |  |  |  |  | Severe and constant anxiety |

1. To what extent do you feel depressed about the breathlessness associated with your health condition?

| **0** | **1** | **2** | **3** | **4** | **5** | **6** | **7** | **8** | **9** | **10** |
| --- | --- | --- | --- | --- | --- | --- | --- | --- | --- | --- |
| None at all |  |  |  |  |  |  |  |  |  | Completely discouraged |

1. To what extent are you pessimistic about the progression of the breathlessness?

| **0** | **1** | **2** | **3** | **4** | **5** | **6** | **7** | **8** | **9** | **10** |
| --- | --- | --- | --- | --- | --- | --- | --- | --- | --- | --- |
| None at all |  |  |  |  |  |  |  |  |  | Completely hopeless |

1. To what extent do you fear dying when you experience breathlessness?

| **0** | **1** | **2** | **3** | **4** |
| --- | --- | --- | --- | --- |
| I don’t think about it |  |  |  | I always think about it. |

## APPENDIX 10. Clinical Undesirable Effects Screening Questionnaire

| **SYMPTOMS**  **(During or after the intervention)** | *Response of the participant (evaluated by investigators)* |
| --- | --- |
| **General well-being** | **Grade**  1 2 3 4  **Comment:** |
| **Skin reactions (itchiness, redness, burning, etc.)** | **Grade**  1 2 3 4  **Comment**: |
| **Muscle contractions (twitching, discomfort, pain, …)** | Grade 1 2 3 4  **Comment:** |
| **Headache** | Grade 1 2 3 4  **Comment:** |
| **Vertigo/dizziness** | **Grade** 1 2 3 4  **Comment:** |
| **Nausea** | **Grade**  1 2 3 4  **Comment:** |
| **Voice changes (hoarseness, aphonia, …)** | **Grade** 1 2 3 4  **Comment:** |
| **Pain (of any type)** | **Grade** 1 2 3 4  **Comment:** |
| **Other** | **Grade** 1 2 3 4  **Comment:** |

For adverse event grading, we followed an adaptation of the Common Terminology Criteria for Adverse Events (CTCAE) 5.0.1 (48)

- GRADE 1: Mild symptoms, with little or no impact on activities. No indication for intervention.
- GRADE 2: Moderate symptoms, slightly more impact on activities. Intervention indicated.
- GRADE 3: Severe symptoms, prevent performance of activities. Intervention or hospitalization indicated.
- GRADE 4: Very Severe symptoms, potentially life-threatening, preventing basic personal care. Intervention indicated to prevent permanent damage/persistent disability/death.
