# Supplementary material for: Non-invasive neuromodulation for alleviating dyspnoea: protocol for a feasibility sham-controlled randomised trial
Source: BMJ Open. 2025 Jul 22;15(7):e103891. doi: 10.1136/bmjopen-2025-103891 (PMC12306373; doi:10.1136/bmjopen-2025-103891)
Supplement: Supplementary file 2 [file bmjopen-2025-103891.reviewer_comments.pdf]

PEER REVIEW HISTORY

BMJ Open publishes all reviews undertaken for accepted manuscripts. Reviewers are asked to complete a checklist review form and are provided with free text boxes to elaborate on their assessment. These free text comments are reproduced below.

ARTICLE DETAILS

Title (Provisional)

Non-Invasive Neuromodulation for Alleviating Dyspnea: Protocol for a Feasibility Sham-Controlled Randomized Trial

Authors

St-Pierre, Joël; Mailhot-Larouche, Samuel; Garand, Geneviève; Vézina, Félix-Antoine; Léonard, Guillaume; Iorio-Morin, Christian; Couillard, Simon

---

VERSION 1 - REVIEW

**Reviewer**	**1**
**Name**	**Delclaux, Christophe**
**Affiliation**	**Université Paris Cité Faculté de Santé, Physiology**
**Date**	**13-Jun-2025**
**COI**	**None**

I've some difficulties with the rational of the study: dyspnea sensation arises when ventilatory capacity cannot face to ventilatory demand, as a protective mechanism that restores homeostasy (demand - capacity balance). For instance, a patient with COPD will stop effort when its ventilatory demand is superior to its capacity. Of course, this alarm signal can be excessive and a patient can learn to manage its dyspnea, using rehabilitation for instance. Thus, the use of neuromodulation should be restricted to settings in whom dyspnea is inappropriate or excessive, in my opinion.

Overall, excepting the indication of neuromodulation, I have no major comment related to the design of this feasibility trial on a restricted number of patients (4 in each group).

The parameters of both cervical transcutaneous vagus nerve stimulator and a facial-trigeminal transcutaneous electric nerve stimulation are specified but are subject of debates and could better be discussed.

Overall, the feasibility is good but the efficacy (that is not the subject of the study) is rather challenging in a disease in which dyspnea relies on a pathophysiological background that does not seem to me the better choice.

VERSION 1 - AUTHOR RESPONSE

Thank you for your thorough reading and thoughtful critique of our manuscript. In response to your comments, we have revised the paper accordingly, addressing the major concern and the other points raised.

The major concern you pointed out has been central to the reflection throughout the development of this project. Although neuromodulation appears interesting for dyspnea relief, we are careful not to advocate for an approach that may create new clinical concerns. Therefore, we have clarified in the Introduction that the goal is to explore neuromodulation as a potential alternative for patients who continue to experience significant dyspnea despite standard “dyspnea-directed” therapies (e.g., opioids, rehabilitation). These additions to the manuscript can be found on pages 6-7.

In response to your comments regarding the intervention parameters, we have added explanations about our choice of initial settings, particularly for the VNS device, and highlighted the flexibility in the parameters for the TENS device. Given the exploratory nature of this study, we emphasize that the parameters are subject to adjustment in order to identify the most promising settings for the intended outcomes. These additions to the manuscript can be found on pages 10-11.

Studying dyspnea in this context presents many challenges, and as the first study in this field, this feasibility trial is primarily designed to assess whether neuromodulation can be reliably studied for its effects on the perception of dyspnea. In other words, our aim is to determine a reliable method to quantify dyspnea relief through neuromodulation, which will guide the design of future large-scale RCTs assessing efficacy.

VERSION 2 - REVIEW

**Reviewer**	**1**
**Name**	**Delclaux, Christophe**
**Affiliation**	**Université Paris Cité Faculté de Santé, Physiology**
**Date**	**07-Jul-2025**
**COI**	

The authors have addressed adequately my few comments
